# Chemical Mechanisms of Nanoparticle Radiosensitization and Radioprotection: A Review of Structure-Function Relationships Influencing Reactive Oxygen Species

**DOI:** 10.3390/ijms21020579

**Published:** 2020-01-16

**Authors:** Douglas Howard, Sonia Sebastian, Quy Van-Chanh Le, Benjamin Thierry, Ivan Kempson

**Affiliations:** Future Industries Institute, University of South Australia, Mawson Lakes 5095, Australia; douglas.howard@mymail.unisa.edu.au (D.H.); Benjamin.Thierry@unisa.edu.au (B.T.)

**Keywords:** radiosensitization, radioprotection, metal nanoparticle, reactive oxygen species, ROS

## Abstract

Metal nanoparticles are of increasing interest with respect to radiosensitization. The physical mechanisms of dose enhancement from X-rays interacting with nanoparticles has been well described theoretically, however have been insufficient in adequately explaining radiobiological response. Further confounding experimental observations is examples of radioprotection. Consequently, other mechanisms have gained increasing attention, especially via enhanced production of reactive oxygen species (ROS) leading to chemical-based mechanisms. Despite the large number of variables differing between published studies, a consensus identifies ROS-related mechanisms as being of significant importance. Understanding the structure-function relationship in enhancing ROS generation will guide optimization of metal nanoparticle radiosensitisers with respect to maximizing oxidative damage to cancer cells. This review highlights the physico-chemical mechanisms involved in enhancing ROS, commonly used assays and experimental considerations, variables involved in enhancing ROS generation and damage to cells and identifies current gaps in the literature that deserve attention. ROS generation and the radiobiological effects are shown to be highly complex with respect to nanoparticle physico-chemical properties and their fate within cells. There are a number of potential biological targets impacted by enhancing, or scavenging, ROS which add significant complexity to directly linking specific nanoparticle properties to a macroscale radiobiological result.

## 1. Introduction

Radiation therapy is currently utilized in more than 50% of all cancer treatments and commonly used in conjunction with chemotherapy or surgery [1]. Although radiation therapy is widely utilized, issues with dose toxicity to healthy tissues on entry and exit from the tumour site limit treatment [2,3]. Therapeutic efficacy of X-ray radiation therapy is dependent on the degree of tumour oxygenation as the ionizing radiation generates reactive species, which impart biological damage. Hence, the inherently hypoxic microenvironment within many solid tumours leads to radioresistance. [1,2,4,5,6]. While it is possible to deliver a radiation dose sufficient to overcome hypoxia and induce cancer cell death, in many instances this is practically not feasible due to dose limitations of normal tissues. An increase in tumour control probability and/or minimization of normal tissue complication probability can improve the therapeutic ratio during radiation therapy [6]. Radiosensitisers that enhance radiation-induced damage to biological targets within tumours can therefore be used to achieve greater tumour responses. A promising avenue for clinical use is metal-based nanoparticles; however, their mechanistic function is a matter of significant debate [7]. There is clear evidence that the nanoparticle physico-chemical properties can contribute to promotion of chemical-based mechanisms, primarily via enhancement of reactive oxygen species (ROS). This review explores the literature with respect to efforts in linking nanoparticle properties to their functional role in impacting intracellular ROS under radiation exposure.

In radiation therapy, cellular damage is caused by both direct and indirect mechanisms. Direct damage occurs when incident photons or particles cleave DNA, leading to apoptosis or necrosis. Indirect damage is primarily caused by interactions of ionizing radiation with oxygen-containing molecules, generating a variety of reactive oxygen species (ROS), including radicals [7,8,9,10,11]. Indirect damages are the predominant mechanism involved in radiation therapy with low linear energy transfer (LET) sources, such as X-rays [12]. ROS are present in all cells and essential for effective cell signalling and function, maintaining cellular homeostasis by use of antioxidants present within the system [13]. Large amounts of these antioxidants, such as glutathione, may be present within cells under hypoxia, decreasing ROS generation and the resulting cellular damages during radiation therapy [14]. The localization of radiation-induced damages and increased levels of ROS generation within cells can overwhelm the antioxidants and redox equilibrium, triggering oxidative stress, biomolecular damages and cell death [15,16]. While some of the biological mechanisms involved in cellular activity and ROS are important to note, they will not be of focus in this review.

Metal nanoparticle-based radiosensitization has received increasingly greater attention in research agendas, literature and clinical use [1,6,7,17,18,19]. The presence of metal nanoparticles within cells/tumour tissues is now well demonstrated to lead to radiosensitization, which can be exploited to improve the overall therapeutic efficacy and reduce side-effects to healthy tissue. Two metal nanoparticle radiosensitisers have progressed to the clinical trialling phase [8,9]. The most advanced of these, hafnium oxide nanoparticles developed by Nanobiotix, has recently achieved European market approval. Nanobiotix is conducting several clinical trials in a variety of cancer types, and have recently reported data from their Phase III trials in soft tissue sarcoma [10,11].

Initially, the concept of metal nanoparticle radiosensitizers was based on physical interactions with ionizing radiation. During X-ray irradiation, photons interact with metal nanoparticles, resulting in primary and secondary physical processes, such as the photoelectric effect, Compton scattering, Auger electron emission, X-ray fluorescence and pair production, with varying cross-section dependencies on atomic numbers and/or X-ray energy [3]. Theoretically, the photo- and Auger electrons contribute to the majority of dose deposition in close proximity to the nanoparticle [12]. Early work placed emphasis on the photoelectric effect and with the cross-section being highly dependent on the atomic number (Z), “high-Z” metals were preferentially investigated [6]. Due to these considerations and the existence of well-defined synthetic routes enabling the reliable preparation of nanoparticles with acceptable biosafety profiles and controlled shapes and sizes, gold nanoparticles have dominated the literature landscape [13,14].

The photoelectric effect exhibits a strong dependence on the energy of the exciting X-ray. However, in vitro studies have shown comparable radiosensitization with MeV X-rays compared to keV X-rays [15] and also with low-Z materials such as carbon [16]. This points out to the fact that other, non-physical, mechanisms are important in producing indirect damage to cancer cells, such as a pronounced increase in ROS generation for both X-ray and particle-based radiation therapies [17]. Further highlighting the importance of ROS is that nanoparticles typically associate with cells in their cytoplasm and/or cytoplasmic vesicles. While increase in DNA damage can be observed in the nucleus [18], the physical dose deposition around a nanoparticle does not extend far enough to have a probable impact within the nucleus. It is therefore clear that the physical mechanisms involved in radiosensitization are insufficient to adequately explain observed biological responses and that better understanding of other mechanisms underlying these effects is required.

A critical aspect of nanoparticle radiosensitization is the mechanism by which they promote the generation of reactive species, especially ROS. Production of ROS are likely to be dependent on many variables and characteristics of the metal nanoparticle, such as size, shape and surface chemistry. The exact mechanisms of ROS generation, and hence their optimization for therapeutic purposes, by ionizing radiation interacting with nanoparticles are yet to be fully elucidated. To address this knowledge gap, this review critically discusses the relevant literature with a focus on discussing the role of metal nanoparticles in altering ROS generation during irradiation, as well as the techniques for measuring ROS in these studies. The goal of this review is to improve understanding of physico-chemical-based mechanisms of radiosensitization and radioprotection in the context of cancer radiation therapy. The majority of studies reported in literature have been conducted in vitro. While in vivo there will be significantly more complex biological processes involved, the basic physical principals enhancing ROS will be maintained. Better understanding of the metal nanoparticle structure-function relationship in enhancing ROS generation will guide the development of more potent agents.

## 2. Nanoparticle Localization, ROS Transport and Cellular Damage

The generally accepted target in radiation therapy is double strand cleavage of cellular DNA by direct or indirect mechanisms [19]. The cell nucleus then intuitively becomes of primary interest to target for targeting nanoparticles [20,21]. Liu et al. utilized gold nanoparticles functionalized with nitroimidazole and a cell penetrating peptide to localise uptake and damage to the cell nucleus and increase oxidants within the cell environment. This resulted in a dose enhancement with a clinically-relevant X-ray source [4]. Typically however, most nanoparticles do not penetrate the nucleus [3,22,23] and there are conflicting reports of DNA damage being associated with nanoparticle radiosensitization. Depending on experimental variables, nanoparticles may [24], or may not [25], increase DSBs. Most ROS are typically short lived and interact within a limited, local environment [26]. This suggests there may be other intracellular targets that induce oxidative stress and cause cell apoptosis; likely targets being endosomes, lysosomes and the mitochondria [27]. This section identifies how ROS can lead to cell death with interest in understanding how, if ROS generation is enhanced, cell death can be achieved with or without ROS causing nuclear DNA damage.

Radiation therapy induces ROS generation in the cell and also alters membrane permeability [28,29]. While ROS plays an important part in proliferation cellular homeostasis, excessive levels disrupt their normal function [30]. Interactions include disrupting the mitochondrial electron transport chain and causing oxidative stress by interacting with nearby biomolecules like lipids, DNA and proteins causing lipid peroxidation, DNA double strand breaks and misfolded proteins [31]. ROS activate signalling pathways both during normal cellular homeostasis and also during radiation therapy [32].

The localization of the metal nanoparticles can potentially also dictate the solubility and stability of the nanoparticles due to pH changes. Intracellular organelles, such as endosomes and lysosomes, are more acidic and may cause instability of metal nanoparticles. Chen et al. proposed that their hafnium-doped hydroxyapatite nanoparticles are localized in or near these organelles and therefore, release hafnium ions into the mitochondrial membrane to generate further ROS due to acidic pH in the tumour cells [33].

**Figure 1:**
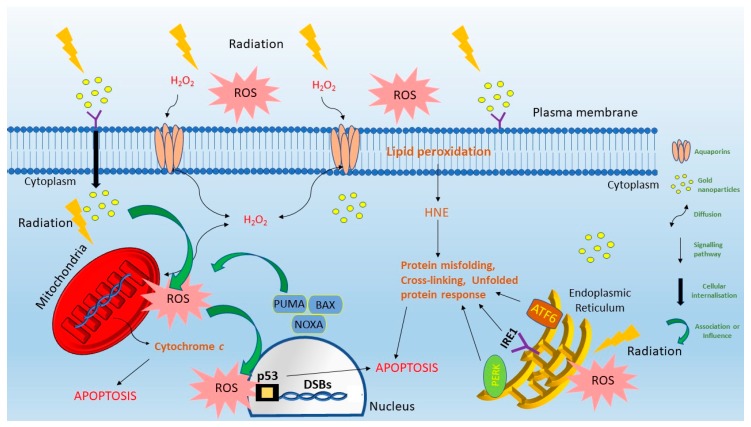
Mechanisms of ROS, generated during exposure to ionizing radiation, leading to apoptosis.

Increasing ROS in cells can lead to apoptosis via a number of mechanisms summarized in Figure 1. Radiation therapy causes DNA damage by ionization and by ROS generation [34]. They induce base oxidation, double strand breaks and single strand breaks [29]. Out of these the double strand breaks causes are most important [29,34]. Double strand breaks activate several sensor proteins like ataxia-telangiectasia and Rad3-related (ATR), ataxia-telangiectasia mutated (ATM) and DNA-dependent protein kinase (DNA-PK) in response to DNA damage repair pathway [35]. These in turn result in the phosphorylation of Chk1 and Chk2 which are checkpoint kinases [35,36]. These activate the p53 during irradiation [29,31]. Cells will attempt to repair double strand breaks most notably via the non-homologous end joining (NHEJ) pathway or by the homologous repair (HR) pathway [37]; however, inadequate repair will result in radiation induced cell death [38].

Radiation and radiation induced ROS leads to lipid peroxidation. This damage is associated with permeability of cell membrane and disruption of transport of molecules across the membrane [31,39]. The poly unsaturated fatty acids of the lipid membrane when peroxidised leads to formation of 4-hydroxy-2-nonenal (HNE) which easily reacts with thiol or amino groups causing cross linking of proteins [31,40]. Greater levels of HNE also triggers unfolded protein response which in turn activates protein kinase R PERK ((PKR)-like endoplasmic reticulum kinase) [41]. This initialises certain transcription factors which trigger the JNK and p38 signalling pathways. This mode of action suggests that HNE might be an upstream regulator between ER stress and radiation induced ROS response [42]. ROS can also trigger sphingomyelinase catalysing hydrolysis of sphingomyelin in cell membranes. This induces production of ceramide [43]. DNA double strand breaks induced by radiation also activates ceramide synthase and is associated with both the intrinsic and extrinsic apoptotic pathways via other signalling cascades [44].

There has been an immense progress in information regarding the production and scavenging of the reactive oxygen species [45]. However, there is not much information regarding their transport from their site of origin, to their place of action or across the cytoplasm within the cell. Their transport is likely to be largely governed by aquaporins and nuclear pore complexes [46,47]. ROS interactions and their transport are strongly involved in intracellular signalling [32,48]. Biological membranes have critical roles in separating organelles and cells, thus separating or compartmentalizing different metabolic and signalling pathways [49]. Thus, altering their permeability can have significant repercussions on the cell.

Small and non-polar molecules can easily diffuse across hydrophobic lipid bilayers [50], though large, polar and charged molecules require channels or transporters for passive diffusion. The similarity between H_2_O_2_ and water (H_2_O) facilitates transport of H_2_O_2_ via aquaporins across membranes [51]. Aquaporins are membrane proteins that function as water channels [52]. Since H_2_O_2_ is similar in size, dielectric properties and can form hydrogen bonds exactly like water, their transport can be attributed to aquaporins in the same way as water molecules [51,53]. A study by Henzler and Steudle, [54] demonstrated that when an aquaporin blocker, like mercury was used in the algae *Chara Corallina*, an accumulation of H_2_O_2_ in the internodal cells was observed. A comprehensive testing of 24 aquaporin isoforms (plants and mammals) and a fluorescence-based assay with intact yeast has provided the first molecular- and genetic- based evidence for the involvement of three aquaporins namely, Haqp8, AtTIP1;1 and AtTIP1;2 in the diffusion of H_2_O_2_ across membranes.

Chantale et al. [55] in their work has demonstrated that an increase in the cytosolic ROS leads to an increase in the nuclear ROS levels. Their study was conducted mainly on human aortic endothelial cells, human vascular smooth muscle cells and human endocardial endothelial cells. They concluded that an increase in cytosolic ROS leads to an increase of ROS within the nucleus after exposure to H_2_O_2_. In further experiments, nuclei were isolated and exposed to H_2_O_2_. Results demonstrated an increase in ROS within the nuclei thus confirming the ability for ROS to translocate through the nuclear membrane. In addition, their results also showed that GSH can reverse this increase of ROS levels in the nuclei. Therefore, the action of GSH is not restricted only to the cytosol. Transport of ROS into the nucleus is not necessary however to have an effect. For example, the nucleus can sense mitochondrial oxidative stress via signalling pathways [56]. Specifically, there occurs a cross-talk or communication between the mitochondria and the nucleus which controls the cell’s response to oxidative stress [56,57]. During oxidative stress, an increase in the respiratory enzyme CDC like kinase 1 (CLK1) is observed in the nucleus [31,56]. These enzymes regulate genes responsible for depleting ROS to maintain mitochondrial homeostasis during oxidative stress. Furthermore, DNA methyl transferase 1 (DNMT1), an enzyme that is regulated by the transcription factors associated with oxidative stress, mediates epigenetic changes in the mitochondria [58].

The p53 gene plays a crucial role during radiation induced oxidative stress to regulate the redox levels in a cell [59]. Intracellular ROS activate p53 which promotes the production of antioxidants that scavenge the ROS within a cell [30]. During high levels of ROS, p53 is activated via the JNK signalling pathway which in turn upregulates the p53-upregulated modulator of apoptosis (PUMA), a prooxidant gene [60]. This gene alters the membrane permeability of the mitochondria, which is associated with p53 dependent apoptosis. The p53 gene not only suppresses the antioxidants related to nuclear factor-E2-related factor 2 (Nrf2), but can directly restrict the Nrf2-mediated transcription [58,61]. Therefore, high levels of ROS contribute to cell apoptosis mediated by the p53 gene [62].

Mitochondria do not have well developed repair systems enabling long-term damage of the mitochondrial DNA when exposed to excessive ROS during radiation therapy [63]. This in turn releases cytochrome *c* stimulating the intrinsic apoptotic pathway [64]. Targeting the mitochondria for this effect can be shown by Fang et al., who conjugated gold nanoclusters with mitochondria-targeting peptides to increase localization of the nanoparticles into the mitochondria, localizing ROS and inducing oxidative stress [65].

The endoplasmic reticulum (ER) is an organelle responsible for synthesizing and folding of proteins. It also responds to radiation and ROS [66]. Cellular stress causes ER dysfunction and triggers signals using ATF6, PERK and IRE1 [67]. Stress to the ER can lead to protein misfolding and unfolding, [68] and when excessively high, signalling leads to induction of apoptosis or autophagy [69,70]. These examples of literature highlight mechanistically how enhancing ROS in a radiosensitization context can enhance cell death either by directly impacting DNA, or other cellular components.

## 3. Mechanisms of Nanoparticle ROS Enhancement

Nanoparticles may enhance formation of ROS during irradiation with ionizing radiation via physical or catalytic processes, or by delivery of oxygen-rich materials. Here, we refer to physical mechanisms as effects associated to locally enhanced physical dose and increase in secondary electron emission. These electrons interact and ionize oxygen-containing molecules in the vicinity of the nanoparticle, generating ROS [71,72].

Catalytic mechanisms are physico-chemical processes that lower the ionization potential of molecules at the nanoparticle-liquid interface or when the nanoparticle acts as an electron donor. The importance of the interfacial water around metal nanoparticles has been investigated with an emphasis on surface chemistry [73,74]. In the work by Liu et al., weak hydroxyl bonds were formed between nanoparticles and adjacent water molecules leading to a lower ionization energy [73]. Secondary electrons with energy lower than that typically required to ionize water, could lead to ionization and hence, nanoparticles could exhibit a catalytic ability to enhance radiolysis and generation of ROS [33,74,75,76].

The third main process is associated to the ability of metal nanoparticles to deliver oxygen-based material to the cancer cells to mitigate hypoxia and increase ROS concentrations. Dissolution of oxygen-based molecules, such as in metal oxides contribute to redox reactions involved in formation of ROS. For example, in the presence of hydrogen peroxide or molecular oxygen, iron oxide nanoparticles undergo Haber–Weiss and Fenton redox reactions to form hydroxyl radicals and superoxide [77,78].

## 4. Types of ROS and Analysis Methods

Within the cell environment, ROS are formed from the reduction of oxygen and are pivotal in naturally modulating cell signalling, cell survival and cell death [26,79]. Significant ROS include free radicals such as hydroxyl (OH^•^), singlet oxygen (^1^O_2_) and superoxide (O_2_**^•^**^−^); the latter can be converted into the non-radical, yet still highly reactive, hydrogen peroxide (H_2_O_2_) [80].

The mitochondria maintains cellular oxidative homeostasis by antioxidants within the microenvironment such as glutathione, catalase and superoxide dismutase [79,81]. A disproportion of superoxide is rapidly reduced into hydrogen peroxide by superoxide dismutase within the mitochondria. Superoxide is a poor oxidant and has a low reactivity toward most biological molecules. Many deleterious effects of superoxide are due to the conversion of superoxide to a more reactive radical, particularly the hydroxyl radical [82]. Hydroxyl radicals can be formed by oxidation of water molecules by iron ions via the Fenton reaction with hydrogen peroxide [83]. These hydroxyl radicals are highly reactive and have a short half-life but can cause severe damage to cells [26,79].

To measure ROS either in solution or in cell studies, different techniques are utilized. Ideally, real-time, in-situ measurements would be performed, however such studies are limited to just a few Raman spectroscopy-based studies. Most ROS have extremely short half-lives, i.e., on the order of nanoseconds for hydroxyl radicals to milliseconds for hydrogen peroxide. As such, it is very challenging to measure ROS directly in real time. It is therefore more practical to use a secondary marker such as fluorescent dyes or radical scavengers with a much longer-lived species such as in colorimetric assays and electron spin resonance respectively. These approaches are described in the rest of this section.

### 4.1. Raman Spectroscopy

Raman spectroscopy can be used for a direct measurement of ROS [84,85], although studies with irradiation are very limited [86,87]. Panikkanvalappil et al. monitored Raman shifts in DNA bands in real-time upon addition of hydrogen peroxide or during irradiation with UV light. In this example, platinum nanoparticles were used to scavenge ROS and reduce DNA damage [87]. Although direct techniques can be used to measure real-time measurement of ROS or their effects, it is a challenge to monitor the effects of clinical irradiation due to the complexity of establishing such measurements within a clinical facility. Establishing real-time measurements with Raman spectroscopy has significant potential for providing valuable mechanistic insight into radiobiological processes and the radiosensitization mechanisms.

### 4.2. Fluorescent Dyes

Fluorescent dye-based assays encompass the most common methods used in radiosensitization literature. Table 1 displays the main fluorescent dyes used in radiosensitization studies and their specificity to reactive species. While the dyes have specificity to different reactive species, they may be weakly sensitive to others, which is an issue regarding identifying specifically which species dominate in imparting a sensitization effect. Fluorescent dyes also face the challenge of degradation of the fluorescence over time; due to exposure to light and the surrounding environment, thus complicating analysis protocols and data interpretation.

2′,7′-dichlorofluorescein diacetate (DCFDA) is the most common dye utilized in radiosensitization and radioprotection literature, with the advantage of being able of measuring ROS either in solution or within cells as the probe is cell-permeable. However, this dye is non-specific and is sensitive to many ROS, including hydrogen peroxide. DCFDA is converted to DCFH by cellular esterases after diffusing into cells or by reaction with a strong base [93]. From this form, DCFH is then oxidized by the ROS into the fluorescent DCF and the fluorescence intensity is analysed using a microplate reader, fluorescence microscope or flow cytometer. While being regularly utilized, DCFDA is not without its limitations. DCFDA suffers from high sensitivity to many species, including reactive nitrogen species. This affinity to nitrogen species may lead to misleading results if wanting to solely measure reactive oxygen species. Low specificity for reactive oxygen species also; may lead to an apparent overestimation of ROS and is more suitable as an indication of oxidative stressors and not for specific species [94,95]. Additionally, DCFDA is only weakly reactive with superoxide anions and is therefore not an effective measurement tool for this specific species [94].

Other dyes that have been used to measure ROS include 3′-(p-aminophenyl) fluorescein (APF), dihydroethidium (DHE), dihydrorhodamine (DHR), singlet oxygen sensor green and 7-hydroxycoumarin. These fluorescent dyes are more sensitive to certain types of ROS. APF and 7-hydroxycoumarin are more sensitive to hydroxyl ions, while DHE and DHR are sensitive to superoxide ions [26]. Another fluorescent dye is nonyl acridine orange, used to target and analyse the oxidative state of the mitochondria. Taggart et al. utilized nonyl acridine orange, and analysed with flow cytometry after treatment to indicate the presence of oxidation of the lipid, cardiolipin, and to show changes in mitochondrial mass and oxidation levels [96]. Gold nanoparticles reduced mitochondrial membrane polarization independent of radiation and lead to an increase in mitochondrial oxidation levels.

Some studies have utilized dimethyl sulfoxide (DMSO) as a radical scavenger in the presence of gold nanoparticles and radiation [97]. Jeynes et al. showed an increased cell survival, determined by clonogenic assay, with samples treated with gold nanoparticles, irradiation and DMSO than the nanoparticles and irradiation alone [3]. DMSO effectively scavenges hydroxyl radicals in cell studies at a concentration that is not negatively affecting cell biology [98]. Use of colorimetric analysis has also been used. Swanner et al. measured the extent of oxidation of cysteine thiols to sulfenic acid using Western blot after nanoparticle and radiation treatment to validate that their 20–30 nm silver nanoparticles depleted glutathione levels through redox reactions, leading to oxidative stress [99,100]. Table 2 presents a list of publications specifically investigating ROS in radiosensitization and radioprotection, identifying the assays, experimental parameters and key observations.

### 4.3. Electron Spin Resonance

Electron spin resonance (ESR) is an analytical technique to detect unpaired electrons and is utilized to measure ROS generation due to the technique’s ability to classify specific radicals [122]. Free radical analysis is made by use of techniques such as spin labelling and spin trapping. Spin labelling compounds utilize an unpaired electron that binds to another molecule and the corresponding magnetic resonance signal is measured by ESR. Spin trapping involves trapping short-lived free radicals and ROS to form longer-lived adducts for ESR analysis [123]. Hydroxyl radicals and other reactive species have a half-life of nanoseconds, compared to the spin trap adducts which can have a half-life of minutes to hours. In radiobiological studies, ESR has been used to primarily measure the scavenging effect of ROS by organic molecules [124,125] and metal nanoparticles [126,127], but nanoparticle radiosensitization has also been explored [112,128,129]. Yin et al. synthesized endohedral metallofullerenol ([Gd@C_82_(OH)_22_]_n_) nanoparticles and showed an increase in ROS scavenging over time and also with an increase in nanoparticle concentration with ESR spin trapping of 5,5-dimethyl-1-pyrroline N-oxide (DMPO) hydroxyl adduct [127]. Yu et al. found that zinc oxide nanoparticles produced ROS themselves, specifically hydroxyl radicals, with the spin trapping compound, DMPO and corroborated this with DCFDA [129]. We are not aware of any studies that utilize ESR spectroscopy with radiation and metal nanoparticles for insights in radiosensitization or radioprotection and is potentially an underutilized analytical method in this field of research.

## 5. Dependence on Metal Content

A large number of metal nanoparticles have been used in radiosensitization studies. Changes in the metal, size, shape and surface functionalization likely play important roles in ROS generation and scavenging. Metals are chosen with respect to biocompatibility, low cytotoxicity or for synergistic abilities, such as gadolinium as a contrast agent for diagnostics [103]. Metals with a high atomic number, particularly gold, have been investigated due to enhancing the physical mechanisms. Metals that have also been commonly investigated as radiosensitizers include platinum, silver, bismuth and hafnium [19,130]. Hafnium oxide-based nanoparticles developed by the company Nanobiotix have achieved European market approval and are being utilized in several clinical trials [11,131]. While hafnium oxide has seen success in terms of clinical development, metals such as platinum and silver have received limited investigation, due to the potential toxicity from dissociation of metal ions [99,132,133]. Gold nanoparticles have instead dominated the field owing to their high biocompatibility, ease and variability of synthesis and ease of surface functionalization [133,134,135].

Metals such as iron and gadolinium have also been of interest as radiosensitizers due to their dual use in providing imaging contrast in MRI [136,137,138]. Furthermore, iron can dissociate and further undergo Haber–Weiss and Fenton reactions to generate, and propagate, ROS [78]. Titanium and copper have also been shown to dissociate metal ions and interact with antioxidants and other enzymes within the cell, enhancing ROS [5,136]. Nanoparticles can also be designed to transport more oxygen to allow for higher ROS generation. In one example by Shao et al., hollow mesoporous silica nanoparticles with sodium percarbonate encapsulated within a hollow core were used as an active oxygen generating nanocarrier. The nanoparticles released hydrogen peroxide upon irradiation, increasing the oxygen content and reactive species within the microenvironment, leading to increased cell toxicity [72].

The focus in radiation studies with metal nanoparticles has typically been in radiosensitization but nanoparticle-based radioprotection has also garnered interest. Ceria nanoparticles have displayed antioxidant properties due to rapid changes in oxidation state between Ce^4+^ and Ce^3+^, displaying their catalase-like activity in less acidic environments, such as the cytoplasm [81,93,105,139]. Li et al. showed that PEG-stabilized ceria nanoparticles as opposed to “naked” ceria nanoparticles, exhibited stronger antioxidant properties. This led to greater scavenging of superoxide and hydrogen peroxide by cerium ions and a decrease in cell DNA damage by ROS. Although nanoparticle uptake was less in comparison to naked nanoparticles, viability of normal liver cells improved under irradiation with PEG-stabilized ceria nanoparticles [139]. Curiously, ceria nanoparticles have demonstrated dual radiosensitization and radioprotection properties, offering protection to healthy tissue while sensitizing cancer cells under irradiation [93]. This was suggested to occur as a result of innate difference in pH between cancer cells and healthy cells [140]. Generally, the cancer cell environment is more acidic and this may trigger ceria nanoparticles to act oxidase-like, whereas, in healthy cells, they act as an antioxidant [141,142]. Further research is required on the underlying mechanisms involved to take advantage of these different metal nanoparticles to determine preferential metals to use in radiosensitization and radioprotection in addition to aspects of potential toxicity.

## 6. Dependence on Size

Nanoparticle size can impact the generation of intracellular ROS during irradiation in several ways. Firstly, size impacts cellular uptake and is influential on receptor mediated endocytosis, one of the primary mechanisms involved in uptake by cancer cells [76,143]. Greater numbers of internalized nanoparticles can result in greater intracellular generation of ROS. However, this also needs to be weighed up against other toxicity issues that may result as a function of size. Several studies have reported maximum uptake with metal nanoparticles that are 10–60 nm in diameter [76,144]. Nanoparticles smaller than 10 nm have shown to have beneficial rapid renal clearance while nanoparticles larger than 100 nm can be captured by the liver [76] or may elicit a phagocytic response [144]. Gold nanoparticles smaller than 2 nm, at concentrations as low at 30 µM have also shown toxicity in vitro, causing cell necrosis and apoptosis after incubation, compared to 15 nm-sized nanoparticles which remained non-toxic up until 6300 µM [145].

Importantly, metal nanoparticle size influences ROS generation. Misawa et al. synthesized citrate coated AuNPs of sizes between 5 and 250 nm in solution and demonstrated that ROS generation is inversely proportional to the diameter of the nanoparticle, indicating the importance of the surface area of the nanoparticle [75]. With smaller nanoparticles, there is higher surface area and therefore, an increased number of active sites for nanoparticle interactions [106] per mass of nanoparticles for catalytic processes to occur. Furthermore, self-absorption of secondary electrons generated under irradiation within the nanoparticles increases with increasing nanoparticle size. Using Monte Carlo modelling, Peukert et al. demonstrated that with increasing nanoparticle size, there is a greater total energy deposition but a reduced dose per gold mass. Gold nanoparticles between 10 and 25 nm were found to maximize the dose enhancement and radiolysis yield [146].

In summary, the size of the nanoparticle is important in balancing nanoparticle biological interactions and maximizing the internalized surface area of nanoparticles to catalyse the generation of ROS under irradiation or number of secondary electrons exiting the nanoparticle volume.

## 7. Dependence on Shape, Structure and Stability

Few studies have focused on the impact of metal nanoparticle shape on ROS generation. Spherical nanoparticles typically have a higher affinity for cellular uptake than other morphologies [110,147,148]. Ma et al. synthesized three different shapes (sphere, rod and spike) of similarly sized, PEG-coated gold nanoparticles to analyse how the change in structure influences ROS generation and radiosensitization. They found gold nanospheres produced a greater amount of ROS, suggesting that it is attributing to radiosensitization over gold nanorods or gold nanospikes. More gold was internalized within the cells containing the nanospheres, indicating the cell’s propensity for higher uptake. Due to an increase in uptake over the other nanoparticles, there was a subsequent increase in ROS generation [110].

Modifying the nanoparticle’s chemical and physical structure also has implications on ROS generation and can be demonstrated by comparing different crystal structures of titanium dioxide. Anatase titanium dioxide generates ROS more readily than a rutile structure due to an increased surface area to volume ratio and stability [5,113,119,149]. In another example, Seo et al. used gadolinium oxide and gadolinium chelate nanoparticles to compare the difference in the atomic bonds and the effects of the “core-inner-valence excitation” under irradiation. They found that because the gadolinium oxide nanoparticle had a weak dipolar coupling between atoms, more ROS was generated compared to the stronger bonds of the gadolinium chelate nanoparticles [115].

A stable dispersion of metal nanoparticles is important for many biological applications and is dependent on nanoparticle shape and surface chemistry. While stable nanoparticles are preferential in biological studies due to biocompatibility [139], some studies suggest that a slight aggregation of nanoparticles increases cell uptake [102,120,150], Adams et al. found the shape of gallium oxyhydroxide nanoparticles was influential on stability and thus on the generation of ROS. Their anisotropic shaped nanoparticles were found to be unstable in aqueous solutions and released gallium ions, leading to an increase in ROS generation, unlike the “orzo” shaped nanoparticle of the same surface chemistry [102].

Dissociation of metal ions after cell uptake can enhance radiosensitization [5,14,99,115,133], either by altering redox chemistry or by enhanced photon-atom interactions. While dissolution of metal ions from nanoparticles may assist with clearance, other questions on biodistribution and potential toxicities arise. The ability of such nanoparticles to distinctly sensitize tumour tissues, rather than healthy tissues to promote the therapeutic ratio during radiation therapy, requires further investigation.

## 8. Dependence on Surface Functionalization

Surface chemistry of metal nanoparticles and the surface interactions govern their physicochemical properties [116]. Most studies functionalize the surface of the metal nanoparticle for stability or synergistic promotion of oxygen content for radiosensitization or scavenging reactive species for radioprotection. The surface functionalization also affects cell uptake and clearance of the nanoparticle.

Coating nanoparticles with polymers increases stability, biocompatibility and subsequently lowers the cytotoxicity of the nanoparticle. Polyethylene glycol (PEG), polyvinylpyrrolidone (PVP) and polyglycerol are nanoparticle capping agents that aid in stability and can operate as binding sites for additional conjugates [134,136]. PEG of different molecular weights are common and simple to functionalize onto the surface of metal nanoparticles, stabilizing their surface [114]. Although this stabilizes the nanoparticle, cell uptake is often decreased [139,151]. Gilles et al. demonstrated a decrease in ROS generation with PEG-coated gold nanoparticles compared with non-capped, citrate gold nanoparticles. They proposed this was likely due to a decrease in uptake and less gold atoms present due to the PEG coating. They suggested stable surface coating of the nanoparticles could disrupt the interface between the metal nanoparticle and oxygen-based molecules in the environment by scavenging ROS by chemical interactions with alcohol and thiol groups on the surface of the nanoparticle [152]. This suggests gold nanoparticles can exhibit antioxidant effects due to their surface chemistry or functionalization [76]. Additionally, Li et al. found that, although there was a reduction in cell uptake with their PEG-coated ceria nanoparticles over uncoated ceria nanoparticles, the PEG coating stabilized the nanoparticles and resulted in more efficient radioprotection [139].

Surface functionalization can be utilized to promote cellular uptake and determine nanoparticle sub-cellular localization. Targeting ligand and peptide conjugation promotes nanoparticle uptake [153] and radiosensitization [4,114,136]. Other surface coatings can also enhance uptake. Klein et al. showed citric or malic-coated superparamagnetic iron oxide nanoparticles displayed increased uptake, leading to significantly higher ROS generation after irradiation than uncoated iron oxide nanoparticles alone. The citric and malic surface coating of the nanoparticles was proposed to be reactive due to the net positive charge, which assisted in catalysing ROS [78]. A positive net surface charge on nanoparticles promotes receptor mediated endocytosis due the cell membrane being negatively charged [108,118,143]. Using silicon nanoparticles in a radiosensitization study with a positively-charged aminosilanized surface functionalization, Klein et al. increased nanoparticle internalization within the mitochondria, inducing oxidative stress, over uncapped silicon nanoparticles, which were found to be localized in the cytoplasm [108]. The effect of different sub-cellular location is discussed in the following section. Cheng et al. hypothesized that a slight electronegative charge on the surface of their gold nanoparticles interacts with superoxide to catalyse production of ROS in vitro [154]. While promoting ROS generation, a negative charge on the surface of the gold nanoparticle could limit nanoparticle uptake.

Surface functionalization of nanoparticles can synergistically increase uptake and transport oxygen-based molecules for increased ROS generation. Nakayama et al. increased ROS generation with titanium peroxide nanoparticles coated with polyacrylic acid, speculating that the coating was being peroxidised by H_2_O_2_, leading to further ROS production [113]. Morita et al. echo this with their own titanium-based nanoparticles, coated with polyacrylic acid and H_2_O_2_. Transportation and diffusion of H_2_O_2_ molecules into the tumour cell environment catalyses the production of H_2_O_2_ and overall ROS in the system [112].

Use of chemotherapeutic drugs have been explored for application with nanoparticle radiosensitization and there are studies that have investigated ROS generation for synergistic enhancement [155,156,157]. Conversely, Vasilieva et al. used nanodiamonds modified with the ROS suppressing chelator, neocuproine. Radicals formed during irradiation were neutralized, leading to an increase in the surviving fraction of cells in a cell colony assay [116].

Antioxidants modulate ROS present within the cells and can be an additional target for nanoparticle radiosensitization. Yong et al. utilized gadolinium and tungsten-based nanoparticles, conjugated with chitosan, to decrease the concentration of glutathione within cells. While gadolinium increased the levels of ROS via physical mechanisms, levels of glutathione were decreased in the cell by a redox reaction with tungsten ions. This decrease of glutathione minimized the antioxidant properties of the cancer cell and radiosensitization occurred [118]. Additionally, silver nanoparticles coated with polyvinylpyrrolidone have shown an affinity to bind and reduce thiol antioxidants within the cellular environment, including glutathione [100,158].

These examples of literature highlight the importance of surface properties not only with respect to stability and cell uptake, but also their synergistic or antagonistic influence on mechanistic action [158].

## 9. Summary

Within this review, metal nanoparticle-based influence on ROS concentrations have been shown to play roles in both radiosensitization and radioprotection. Many studies investigate the effects of the nanoparticle structure and surface functionalization for nanoparticle uptake and radiosensitization during radiation therapy. The focus on the role of ROS as an important and exploitable mechanism is steadily growing. There is great scope to fundamentally understand, and optimise, the nanoparticle structure-function relationship with respect to maximizing the effects of ROS generation in radiosensitization. Critical to achieving this is reliable and robust analytical methods. The most utilized fluorescent dye approaches lack specificity, real-time measurements and rely on convoluted mediatory processes which introduce artefacts during data acquisition and interpretation. Real-time, direct measurements of reactive species in biologically relevant environments, encompassing adsorbed biomolecules, pH ranges and redox conditions are highly desirable for translation of a lab-based optimized nanoparticle to providing therapeutic efficacy.

Many different metal nanoparticles with different sizes, compositions and surface functionalization are utilized in radiosensitization studies. Because of these variables, it is difficult to compare and identify common underpinning mechanisms of ROS generation. Significant challenges exist even in comparing one nanoparticle formulation to another as cellular internalization and fate are highly heterogeneous within, and between, cell populations and types. Even if different cell populations have statistically comparable average uptake of nanoparticles, the degree of heterogeneity between cell populations can still be statistically different [159]. There is great scope for fundamental investigation into the physico-chemical mechanisms associated with ROS generation/scavenging for optimizing potential clinical use of metal nanoparticle radiosensitizers.

## Figures and Tables

**Table 1:** Summary of fluorescence dyes assays for reactive oxygen species (ROS) measurement and their specificity.

Assay	Specificity
2′,7′-dichlorofluorescein diacetate (DCFDA)	Non-specific for most ROS or nitrogen species [88,89]
7-hydroxycoumarin	Hydroxyl radical from hydrogen peroxide [89]
Dihydrorhodamine (DHR)	Superoxide radical, peroxynitrite anion and hydroxyl radical [90,91]
3′-(p-aminophenyl) fluorescein (APF)	Hydroxyl radical, hypochlorite or peroxynitrite anion [88,89]
Dihydroethidium (DHE)	Superoxide radical and hydroxyl radical [89,91]
Singlet oxygen sensor green	Singlet oxygen [92]
MitoSOX	Superoxide radical [91]

**Table 2:** Summary of reported ROS measurements and key observations regarding ROS using fluorescent dyes.

Authors	Type of Nanoparticle and Size	Measurement Method	Radiation Dose	Key Observations
Abdul Rashid et al. [101]	Gold nanospheres, superparamagnetic iron oxide NPs, platinum nanodiamonds and bismuth oxide nanorods Size: 1.9 nm, 15 nm, 42 nm, 70 nm respectively	DCFDA with HCT 116 cell line	4 Gy from a 150 MeV proton beam	In order of sensitization enhancer ratio; SPIONs < AuNPs < PtNDs < BiNRs. This was reflected in ROS generation and suggested to be the main variable between different NPs
Adams et al. [102]	Gallium oxyhydroxide in an anisotropic and “orzo” shape Size: 53 nm and 49 nm respectively	DCFDA in vitro with PC12 cell line	Up to 10 Gy from a 6 MeV LINAC	Generation of ROS was related to the stability and structure of NPs. The less stable the NP, the greater ROS generation due to an increased number of metal ions and chemical interactions
Bouras et al. [103]	Superparamagnetic iron oxide conjugated with cetuximab Size: 10 nm core	DCFDA in vitro with U87MG cell line	10 Gy from a 320 keV X-ray source	Cetuximab coated iron NPs had higher internalization and ROS generation compared to non-coated NPs
Chen et al. [33]	Hafnium-doped hydroxyapatite nanocrystal Size: 100 nm	DCFDA in vitro with A549 cell line	5 Gy from a 662 keV gamma source	Radiolysis enhancement due to physical mechanisms. Suggested hafnium ions near intracellular organelles to promote ROS generation
Chen et al. [93]	Ceria coated with neogambogic acid Size: 3–5 nm before coating	DCFDA in vitro with MCF-7 cell line	6 Gy from a 6 MeV LINAC	Ceria NPs promoted autophagy of tumour cells, while also contributing to radioprotection by inhibiting ROS due to Cs^4+^
Choi et al. [104]	Pegylated gold NPs Size: 20 nm	Dihydrorhodamine in vitro with MDA-MB-231 cell line and in vivo in a murine model	2–10 Gy from 320 kV X-ray source	Gold NPs functionalized with dihydrorhodamine was used to analyse ROS on the surface of the NP.
Colon et al. [105]	Cerium oxide NPs Size: Possibly 3–5 nm or 10–50 nm aggregates	DCFDA in vitro with CRL-1541 cell line	20 Gy from a 160 keV X-ray source	Increased radioprotection by scavenging and regulating ROS by the increased ratio of Ce^4+^ and upregulation of superoxide dismutase 2
Fang et al. [65]	Peptide templated gold nanoclusters Size: 3 nm	DCFDA in vitro with MCF-7 cell line	4 Gy from a 160 keV X-ray source	Increased ROS generation and radiosensitization when NPs were targeted to mitochondria
Gilles et al. [74]	Uncoated gold NPs Size: 32.4 nm	7-hydroxycoumarin in solution	15 Gy from a 17.5 keV X-ray source	Physical mechanisms do not govern radiosensitization. Physico-chemical mechanisms and the interfacial water around NPs is important for ROS production and radiosensitization
Higgins et al. [106]	Titania NPs loaded with gold Size: 6.5 nm and 21.6 nm	Methylene Blue degradation in solution	35 Gy/min from a 225 kV X-ray source	NPs displayed radiosensitization by radical generation. Smaller NPs have increased surface area and more catalytic sites for chemical interactions
Jeynes et al. [3]	Gold NPs conjugated with fetal bovine serum or TAT peptide Size: 60 nm and 80 nm respectively	DMSO in vitro with RT112 cell line	5 Gy from a 250 kVp X-ray source and 5 Gy from a 3 MeV proton source	During X-ray irradiation with NPs, DMSO scavenged ROS. This was not seen with a proton experiment
Jiang et al. [107]	Copper oxide NPs Size: 5.4 nm	DCFDA with MCF-7 cell line	6 MV X-ray source	Copper oxide NPs contributed to ROS generation and autophagy
Khalil et al. [97]	Citrate-coated gold NPs Size: 9 nm, 21 nm and 30 nm	DMSO and 2-amino-2-hydroxymethyl-1-3-propanediol in water	11–89 Gy with a 1.5 keV cathode source	H_2_O_2_ was crucial in production of hydroxyl radicals, mediated by gold NPs. Radical scavengers confirmed higher ROS production with smaller gold core
Klein et al. [108]	Silicon coated with amino-silane Size: 1 nm	DCFDA in vitro with MCF-7 and 3T3 cell lines	3 Gy from a 120 keV X-ray source	NPs enhanced mitochondrial membrane depolarization, provoking oxidative stress
Klein et al. [78]	Superparamagnetic iron oxide NPs uncoated and coated with citric or malic acid Size: 9–20 nm uncoated, 7–17 nm with citric acid coat and 6–16 nm for malic acid coat	DCFDA in vitro with MCF-7, Caco-2 and 3T3 cell lines	1 Gy or 3 Gy from a 120 keV X-ray source	Internalization of the NPs into the mitochondria provoked oxidative stress under irradiation
Liu et al. [4]	Gold NPs with different coatings Size: 13 nm gold	DCFDA in vitro with A431 cell line	10 Gy from a 6 MeV LINAC	NPs released nitrite ions upon irradiation to increase ROS generation due to nanoparticle coating
Lu et al. [109]	La_2_O_3_, CeO_2_, CeO_2_-Gd, Nd_2_O_3_, Nd_2_O_3_-Si, Gd_2_O_3_ Size: <100 nm for all	DCFDA with U-87 MG and Mo59K cell lines	3 Gy from 250 keV source	Cell lines responded differently to NPs incubation and irradiation. Gd and Ce based NPs generated ROS
Ma et al. [110]	Gold nanospheres, nanospikes and nanorods Size: 53.2 nm nanospheres, 54.0 nm nanospikes and 50.2 nm nanorods	DCFDA in vitro with KB cell line	4 Gy from a 6 MeV LINAC	Shape affected internalization. Unclear if increases in ROS generation were shape dependent or due to difference in internalization. Spheres were the most effective
Ma et al. [111]	FePt NPs in nanosheets Size: 3.05 nm particles and 500 nm nanosheet	DCFDA with H1975 cell line	4 Gy from a 204 kV photon beam	The nanosheet inhibited cell proliferation and increased ROS generation. Once in the cytoplasm, FePt NPs were internalized in the mitochondria and lysosome
Misawa et al. [75]	Citrate-coated gold Size: 5–250 nm	3′-(p-aminophenyl) fluorescein and dihydroethidium respectively and in solution	Up to 10 Gy from a 100 keV X-ray source	ROS generation was proportional with the inverse of the diameter of the nanoparticle
Morita et al. [112]	Polyacrylic acid-modified titanium dioxide with H_2_O_2_ Size: 124 nm	3′-(p-aminophenyl) fluorescein in solution	Up to 18 Gy from an 80 keV X-ray source	H_2_O_2_ bound to surface and gradually released from nanoparticle surface, adding ROS
Nakayama et al. [113]	Titanium peroxide with coating of polyacrylic acid Size: 50–70 nm	3′-(p-aminophenyl) fluorescein, DCFDA and dihydroethidium. Measured in solution and in vitro with MIA PaCa-2 cell line	Up to 30 Gy from a 150 keV X-ray source	Nanoparticle coating peroxidised into H_2_O_2_, catalysing ROS generation
Nicol et al. [114]	Gold NPs functionalized with peptides Size: 28.7 nm before peptides and 45.9 nm after	DCFDA in vitro with MDA-MB-231 and MCF-7 cell lines	2 Gy from a 160 keV X-ray source	Nanoparticle coating inhibited SOD-2 expression and promotes cellular uptake, leaving cells susceptible to increased levels of ROS
Seo et al. [115]	Gadolinium oxide and gadolinium-chelate NPs Size: 40–45 nm	Dihydrorhodamine in vitro with CT26 cell line	Up to 15 Gy from a 45 MeV proton source	Gd ions from Gd-Gd de-excitation promoted ROS generation for radiosensitization
Shao et al. [72]	Hollow mesoporous silica NPs with sodium percarbonate in the cavity and coated with polyacrylic acid Size: 290 nm with 80 nm core	DCFDA in vitro with ZR-75-30 cell line	Unknown dose from a 60 keV X-ray source	NPs transported sodium percarbonate to the cancer microenvironment, increasing oxygen and generation of ROS
Taggart et al. [96]	Aurovist™ gold nanoparticles. Size: 1.9 nm	Nonyl-Acridine Orange in vitro with MDA-MB-231 and DU145 cell lines	2 Gy with a 225 kV X-ray generator	Gold NPs and irradiation increased levels of ROS, leading to reduced mitochondrial membrane polarization
Vasilieva et al. [116]	Nanodiamonds conjugated with neocuproine Size: 6 nm	DCFDA in vitro with HepG2 cell line	3 Gy from a ^137^Cs gamma source	NPs scavenged ROS but mechanisms are not well known
Wu et al. [117]	Silver coated with polyvinylpyrroliodone Size: 15.38 nm	DCFDA in vitro and MitoSOX (mitochondrial probe) with U251 cell line	No irradiation source used for ROS generation	Silver NPs increased inhibition of protective autophagy and ROS generation was increased
Yong et al. [118]	Gadolinium-containing polyoxometalates-conjugated chitosan Size: 30 nm	DCFDA in solution and in vitro with BEL-7402 cell line	2 Gy from an unknown X-ray source	NPs reduced glutathione levels by redox reaction. Reduction of antioxidants lead to increased levels of ROS and oxidative stress
Youkhana et al. [119]	Anatase titanium oxide coated with aminopropyl trimethoxysilane Size: 30 nm	DCFDA in vitro with HaCaT and DU145 cell lines	15 Gy and 14 Gy from a 6 MeV LINAC	ROS generation was dependent on the nanoparticle concentration
Yu et al. [120]	Selenium NPs coated with PEG Size: 500 nm	DCFDA in solution	8 Gy from an unknown X-ray source	ROS generation using DCFDA was time dependent, decreasing intensity after 40 min. NPs contributed to ROS generation and degraded in cells
Zhou et al. [121]	Bismuth heteropolytungstate (BiP_5_W_30_) nanocluster Size: 1.5 nm	Terephthalic acid in solution. ELISA kit with human hydroxyl radical capture antibody in HeLa cell line. DCFDA was also used.	50 kV with unknown X-ray source	Nanocluster promoted radiosensitization through physical and physico-chemical mechanisms. Depletion of glutathione by redox reactions, further promoting oxidative stress
